# Characterizations of emulsion gel formed with the mixture of whey and soy protein and its protein digestion under in vitro gastric conditions

**DOI:** 10.1016/j.crfs.2023.100674

**Published:** 2023-12-31

**Authors:** Yu Cheng, Aiqian Ye, Harjinder Singh

**Affiliations:** a School of Food and Biological Engineering, Jiangsu University, Zhenjiang, Jiangsu, 212013, China; b Institute of Food Physical Processing, Jiangsu University, Zhenjiang, Jiangsu, 212013, China; c Riddet Institute, Massey University, Private Bag, Palmerston North 4442, 11 222, New Zealand

**Keywords:** Composite gel, Rheological properties, Hardness, Microstructure, Dynamic digestion

## Abstract

Partially replacing animal proteins with plant proteins to develop new products has much attention. To get knowledge of their application in emulsion gels, heat-induced composite protein emulsion gels were fabricated using the mixtures of whey protein isolate (WPI) and soy protein isolate (SPI) with the final total protein concentration of 10% (w/w). The water holding capacity (WHC), mechanical and rheological properties and microstructure of mixed protein emulsion gels prepared at different WPI to SPI ratios (100:0, 90:10, 70:30, 50:50, 30:70, 10:90, 0:100, w/w) were investigated. The ratios of WPI to SPI showed little effect on the WHC of the mixed protein emulsion gels (p > 0.05). Increasing the ratio of SPI decreased the hardness and storage modulus (G′) of mixed protein emulsion gels, whereas the porosity of mixed protein emulsion gels in the microstructure increased, as shown by CLSM. Both β-lactoglobulin and α-lactalbumin from WPI and 7 S and 11 S from SPI participated in forming the gel matrix of mixed protein emulsion gels. More protein aggregates existed as the gel matrix filler at the high soy protein levels. Interestingly, the G′ of mixed protein emulsion gels at the WPI to SPI ratio of 50:50 was higher than the sum of G′ of individual WPI and SPI emulsion gels. The whey protein network predominated the gel matrix, while soy protein predominated in the active filling effect. When subjected to an in vitro dynamic gastric digestion model, soy protein in the gels (WPI:SPI = 50:50) degraded faster than whey protein during gastric digestion. This study provided new information on the characteristics of composite protein emulsion gel fabricated with the WPI and SPI mixture.

## Highlights

• Increasing SPI content decreased the hardness and G′ of the composite gels
• Whey protein-soybean protein co-aggregation occurred in the gel formation
• G′ of composite gels (WPI/SPI = 50/50) was higher than that of the sum of whey protein emulsion gels (WPI/SPI = 50/0) and soy protein emulsion gels (WPI/SPI = 0/50)
• Soy protein degraded faster than whey protein during gastric digestion.

## Graphical abstract

**Figure:**
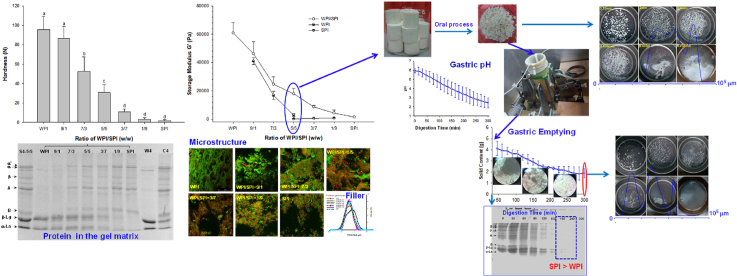

## 1 Introduction

Gels, regarded as soft solids, are present in many foods. As one of the critical forms of food gels, protein gels have been investigated extensively. Most protein gels were made using animal protein. As the consumption of animal proteins produces more carbon emissions than plant proteins, partially replacing animal proteins with plant proteins is considered one of the effective methods to reduce carbon emissions. Recently, protein gels consisting of mixtures of animal and plant proteins were investigated, such as pea protein and whey protein (Kornet, et al., 2021), pea protein and casein (Nascimento, et al., 2023), soy protein and whey protein (McCann, et al., 2018; Xia et al., 2022), potato protein and whey protein (Andlinger, et al., 2021; H. Zhang et al., 2023), soy protein and sodium caseinate (Martin, et al., 2016), soy protein and myofibrillar protein (Lin, et al., 2019; Niu et al., 2018). Since whey protein and soy protein are one of the extensively used commercial food proteins, whey and soy protein composite gels have got much attention. A few studies have been done on mixing soy protein and whey protein gels (Comfort and Howell, 2002; Jose et al., 2016; McCann et al., 2018; Roesch and Corredig, 2005; Xia et al., 2022). However, little has been conducted on the composite soy protein and whey protein emulsion gels (Zhang et al., 2022).

Food protein emulsion gels, made up of emulsion filled protein gel and protein-stabilized emulsion gel, are an essential form of protein gels in food products like yoghurt, cheese and sausage (Dickinson, 2012). Lipid is incorporated into the gel system and can deliver lipophilic components, such as flavourings, essential oils and fat-soluble bioactive compounds (Torres, et al., 2016). Moreover, food gels are often used as model foods because of their lower compositional and structural complexity than natural foods (Vilgis, 2015). Previous research has introduced the formation of protein emulsion gels using different methods with different proteins from a single source (Dickinson, 2012). Nevertheless, most of the recently published work still focused on single-source protein emulsion gels, including whey protein (Lu, et al., 2021; Mao et al., 2021), soy protein (Geng, et al., 2022; K. Luo et al., 2019) or mixing protein and polysaccharide emulsion gels (He, et al., 2021; Yang et al., 2021). Besides, what has been done on emulsion gels with dual proteins was to prepare emulsion gel by mixing one protein and pre-emulsified emulsion made with other proteins (Lu et al., 2021; Mao et al., 2021). However, little research has been conducted on mixing proteins from different sources as emulsifiers and matrices to prepare emulsion gels.

The effect of food structure on the digestion of macronutrients and the delivery of nutrients during the gastrointestinal tract was concerned (Guo, et al., 2017; Lin et al., 2022; Lorieau et al., 2018; Singh et al., 2015; Wang et al., 2019). Significant progress has been made in the digestion of food emulsions (Acevedo-Fani and Singh, 2022; Singh and Ye, 2013). Moreover, several studies on the digestion of protein gels (Guo, et al., 2020; Q. Luo et al., 2017; Nyemb et al., 2016) and protein-based emulsion gels (Cheng et al., 2021; N. Luo et al., 2023; Mennah-Govela and Bornhorst, 2021; Peng et al., 2021) have been conducted in recent years. However, little is known about the digestion of soy and whey protein emulsion gels with higher soy protein content.

Therefore, the present research aims to explore the mechanical and structural properties of mixed soy protein and whey protein emulsion gels. Furthermore, in vitro gastric digestion behaviours of mixed soy protein and whey protein emulsion gels were investigated.

## 2 Materials and methods

### 2.1 Materials

Whey protein isolate (WPI 895, protein content ≥90%, moisture content≤ 4.5%,w/w) was obtained from Fonterra Co-operative Group Ltd. (Auckland, New Zealand), and soy protein isolate (SPI) (PRO-FAM ® 891, protein content ≥90%, moisture content≤ 6%, w/w) was obtained from ADM Ltd. (Hamilton, New Zealand). Refined soybean oil was purchased from Davis Trading Company (Palmerston North, New Zealand). Pepsin from porcine gastric mucosa (Sigma P7000, 250 units/mg solid) was purchased from Sigma-Aldrich Corporation (St. Louis, MO, USA). All other chemicals were of analytical grade. Milli-Q grade water (Milli-pore Corp., Bedford, MA, USA) was used for all experiments.

### 2.2 Preparation of the mixing protein solution

Whey protein and soy protein stock solutions were prepared separately by dissolving WPI and SPI powder in 20 mmol/L phosphate buffer (PB) at pH 7.0. The whey protein and soy protein stock solutions were dispersed for 4 h and stored at 4 °C until use. The hybrid protein solutions were prepared by mixing whey protein and soy protein stock solution at 10:90, 30:70, 50:50, 70:30 and 90:10 (w/w) to reach a final protein concentration of 12.5 % (w/w).

### 2.3 Preparation of emulsions

The coarse emulsions containing 80 % (w/w) protein solutions and 20 % (w/w) soybean oil were prepared using a high-speed mechanical mixer (L5M, Silverson, MA, USA) at 12,000 rpm for 2 min. Then, the coarse emulsions were passed through a two-stage valve homogenizer (HomoLab2, FBF ITALIA, Parma, Italy) twice at the pressure of 300 bar for the first stage and 30 bar for the second stage. Sodium chloride was added to the fine emulsions at a concentration of 50 mmol/L.

### 2.4 Preparation of emulsion gels

Ten-gram emulsions were then put into cylindrical tubes and heated at 95 °C for 30 min in a water bath. After heating, the gels were cooled and stored at 4 °C for 16 h before further use.

### 2.5 Particle size distribution and zeta-potential of emulsion particles

Particle size distribution of emulsion particles was measured by Mastersizer (2000) (Malvern Instruments, Worcestershire, UK). Moreover, the zeta-potential of emulsion particles was measured by Zetasizer nano ZS (Malvern Instruments, Worcestershire, UK). Gel samples were dissolved in 5 % (w/v) SDS containing 50 mmol/L mercaptoethanol, followed by shaking at 50 °C for 8 h. The size of restructured emulsions was determined using Mastersizer 2000 to estimate the size of the oil particle incorporated in the gel matrix.

### 2.6 Distribution of proteins in emulsions and gels

The protein compositions distributed in the emulsions and gels were prepared by centrifuge and demonstrated using sodium dodecyl sulphate polyacrylamide gel electrophoresis (SDS-PAGE) according to the procedure of Ye and Singh (2000).

The emulsions were centrifuged at 30,000 g for 45 min at 20 °C. Three compositions were obtained, including cream, serum and sediment. The cream layer was carefully removed from the centrifuge tube and dehydrated in filter paper. Then, the dehydrated cream was dispersed in 20 mmol/L PB (pH 7.0) and re-centrifuged at 30,000 g for 45 min at 20 °C, followed by suspending in the same PB (equivalent in volume to the initial serum phase) with gently stirring. The subnatant was carefully removed using a syringe, re-centrifuged at 30,000 g for 45 min at 20 °C, and then passed through 0.45 μm filters. The sediment was dispersed in 20 mmol/L PB (pH 7.0) and re-centrifuged at 30,000 g for 45 min at 20 °C. That was followed by suspending in the same PB (equivalent in volume to the initial serum phase) with gentle stirring.

The gels were centrifuged at 10,000 g for 20 min at 20 °C. The liquid (protein not in gel) excluded from gels was collected and passed through 0.45 μm filters. The solid gels were dissolved in 5 % (w/v) SDS containing 50 mmol/L mercaptoethanol, followed by shaking at 50 °C for 8 h to form the restructured emulsions.

A certain amount of restructured cream, subnatant and precipitant from emulsions and the liquid and restructured emulsions from gels were respectively mixed with buffer (0.5 M Tris, 2% SDS, 0.05% mercaptoethanol, pH 6.8) at equal volume, following by boiling for 5 min and centrifuging at 10, 000 g for 10 min. The samples (20 μL) in the supernatant were loaded to SDS−PAGE to identify the composition of proteins in the emulsions and gels using a 4% acrylamide stacking gel and 12% acrylamide separation gel (Laemmli, 1970). The protein concentration was not standardized, so the relative distribution of proteins between the samples could be compared. The running voltage was set at a constant 120 V, and the running time was approximately 1.5 h. Then, the gels were stained for 60 min using a Coomassie brilliant blue R-250 solution with gentle shaking. The gels were detained with a solution containing 10% acetic acid and 10% isopropanol and scanned using a Molecular Imager Gel Doc XR system (Bio-Rad, Hercules, CA).

### 2.7 Measurement of rheological properties

Dynamic rheological measurements were performed by a rheometer (AR, 2000; TA Instruments, New Castle, DE, USA) with a concentric cylinder measuring system in a linear viscoelastic region (the strain of 0.5 %) at a frequency of 1 Hz. Briefly, emulsion samples covered with 1 mL mineral oil were heated from 25 to 95 °C at a rate of 5 °C/min and held at 95 °C for 30 min, followed by cooling from 95 to 25 °C at a rate of 5 °C/min and holding at 25 °C for 10 min. The apparent viscosity of the emulsions was determined using parallel plate geometry with a diameter of 40 mm at the shear rate of 0.1–100 s^−1^ at 25 °C.

### 2.8 Measurement of gel hardness

The formed gels (25 mm diameter and 30 mm high) were exposed to a TA-XT2 texture analyzer (TA instruments, Delaware, USA) and compressed with a cylindrical probe (50 mm diameter). Compression was done at a strain of 30 % with a crosshead speed of 1 mm/s.

### 2.9 Water holding capacity (WHC)

The gels were taken off the tubes and weighed. The weighed gels were packed with filter paper and placed in centrifuge tubes with tissue papers at the bottom to keep water. Samples were then centrifuged at 2000 g for 10 min at room temperature. The samples were weighed again after removing the filter paper. The WHC of the gel was calculated as

$$WHC\left(\%\right)=100\times \left(W_{1\;}-W_{2}\right)/W_{I}$$

where *W*_1_ and *W*_2_ are the weights of gel samples before and after centrifugation.

### 2.10 Confocal scanning laser microscopy (CLSM)

The gel samples were cut into small pieces and stained overnight with fast green (1.0 % (w/v) in water) for protein and Nile red (0.5 % (w/v) in acetone) for oil. Then, the stained gels were placed on a slide and covered with a cover slip. Furthermore, the images were obtained by sequential scanning with CLSM (Leica Microsystems Inc., Heidelberg, Germany) using a 100-magnification oil immersion lens. The excitation wavelengths for protein and oil were 633 nm and 488 nm, respectively.

### 2.11 In vitro digestion of protein emulsion gels

The gels were mechanically ground by a mechanical grinder (MultiGrinder II EM0405, Sunbeam, Australia) for 1 s, according to Guo et al. (2014). The particle size distribution of gel fragments was determined by sieving. The simulated gel boluses were prepared by mixing 200 g of gel fragments with 40 mL simulated saliva fluid (SSF) and warmed at 37 °C for 2 min. Then, the simulated gel boluses were fed to a dynamic human gastric simulator (HGS) model designed by Kong and Singh (2010) for in vitro gastric digestion, as described by Guo et al. (2014). Before digestion, 70 mL of simulated gastric fluid (SGF) containing pepsin (3 g/L) was loaded to mimic the fasting condition of the human stomach. Then, digestion was started immediately at 37 °C. The 1.25**×** concentrated SGF and pepsin solution (3 g/L) were pumped into the HGS separately, and their inflow rate was 2.0 and 0.5 mL/min, respectively. SSF and SGF were prepared according to the protocol described by Minekus et al. (2014).

The digesta were removed from the bottom of the HGS at 45 mL/15 min from 30 min with the gastric emptying rate of 3.0 mL/min during the digestion time of 300 min. The protein breakdown of WPI and SPI in gastric chyme during stomach processing was determined by the SDS-PAGE. Furthermore, gastric digesta emptied at different times was dried overnight at 105 °C, and the solid content of emptied gastric digesta was measured.

### 2.12 Statistical analysis

All the experiments were repeated at least three times on different days using fresh samples, except that the trials on digestion were repeated twice on different days. The data were analyzed with one-way ANOVA using SPSS 21 statistics software. Tukey HSD's test was used to compare the differences between means.

## 3 Results and discussion

### 3.1 Properties of the emulsions

#### 3.1.1 Particle size of the emulsions

**Fig. 1:**
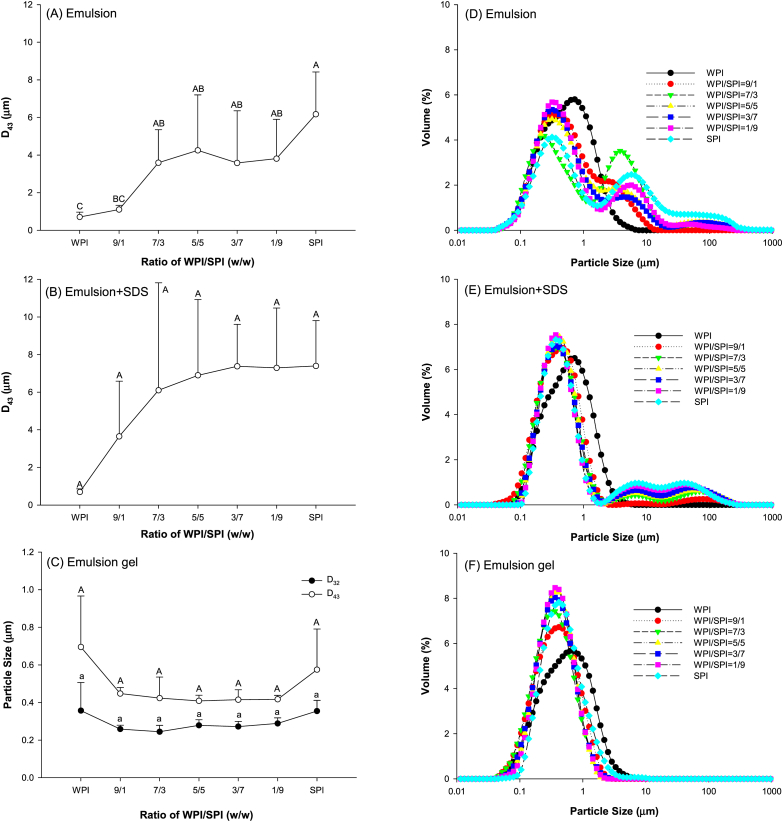
Average particle size and particle size distribution of emulsion samples (A and D) (n = 4), emulsion samples diluted with 1.0 wt% SDS (B and E) (n = 2) and emulsion gel samples dissolved using 50 mM β-ME and 5.0 wt% SDS (C and F) (n = 2).

The particle size and particle size distribution of emulsions prepared with different ratios of WPI and SPI mixtures are shown in Fig. 1A and D. The results showed that the average particle size (d_43_) of emulsions prepared using SPI was higher than those prepared using WPI (p < 0.05). In addition, partially replacing WPI with SPI increased the d_43_ of the emulsions. As shown in Fig. 1D, emulsions prepared with WPI displayed monomodal peaks in particle size distribution, while emulsions prepared with WPI and SPI mixtures displayed multiple peaks. Although the incorporation of SPI resulted in lower particle size in the range of 0.1–1 μm, two new peaks with the particle size distribution from 1 to 10 μm and 10–100 μm were observed. Moreover, those particle size distribution curves were similar to emulsions prepared with SPI.

**Fig. 2:**
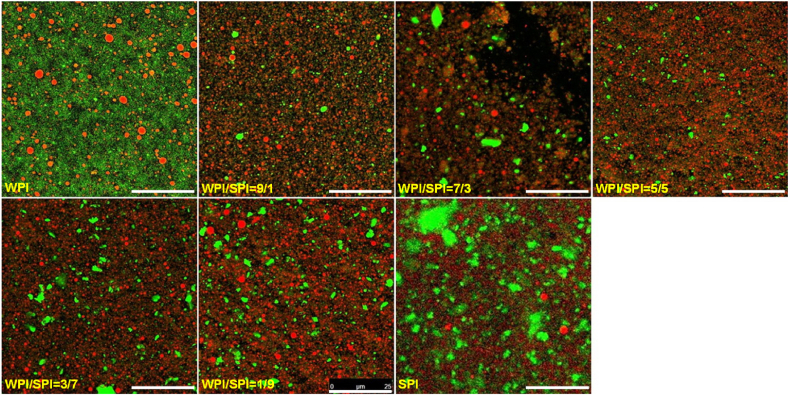
CSLM images of emulsions prepared with the mixtures of WPI and SPI at different ratios (Scale bar: 25 μm).

The particles distributed between 1 to 10 μm and 10–100 μm could be made up of protein aggregates. To clarify that, the emulsion samples were diluted using 1% SDS to disturb the possible flocculation of emulsion droplets and aggregation of proteins. Then, the particle size of those emulsions was determined. As displayed in Fig. 1B, the average particle size d_43_ of those emulsions did not show a significant difference (p > 0.05). Interestingly, although the peak area in the range from 1 to 10 μm decreased remarkably, the peaks in the range from 1 to 10 μm and from 10 to 100 μm did not disappear (Fig. 1E). It suggested the existence of the covalently crosslinked protein aggregates in the emulsions because SDS could not break the covalent bonds. It has been shown that the thermal process of the soy and whey protein mixtures resulted in the formation of aggregates in the size of 10–100 nm (Xia et al., 2022). High-pressure valve homogenization during emulsion preparation might raise the temperature of the emulsions, resulting in the thermal process. Higher soy protein content in the mixture resulted in a larger area for those peaks. The aggregates might contain soy protein. That could be confirmed by the results of the microstructure of the emulsions using CLSM. As shown in Fig. 2, small emulsified oil droplets with a particle size distribution in the range of 1–10 μm were displayed in the microscopy images of emulsion samples. On the contrary, protein aggregates (green particles) with a size from 10 to 100 μm were observed in these images. Thus, the peak with a size from 10 to 100 μm in the particle size distribution curve was considered to be protein aggregates.

The filling effect of emulsion droplets is related to their size (Dickinson, 2012). Therefore, the particle size of emulsified droplets in gels was assessed with emulsion gel samples dissolved using 50 mM β-ME and 5.0 wt% SDS. As displayed in Fig. 1C, the average particle sizes d_43_ and d_32_ of filling emulsified droplets showed little difference (p > 0.05), respectively. It suggested that the ratio of WPI/SPI did not have a significant effect on the average particle size of emulsion droplets. As expected, the peaks in the range from 1 to 10 μm and from 10 to 100 μm disappeared (Fig. 1F). The solvent of 50 mM β-ME and 5 wt% SDS can destroy the covalent and non-covalent bonds of protein aggregates. It might dissociate the protein aggregates formed in the gel matrix.

#### 3.1.2 Zeta-potential

**Fig. 3:**
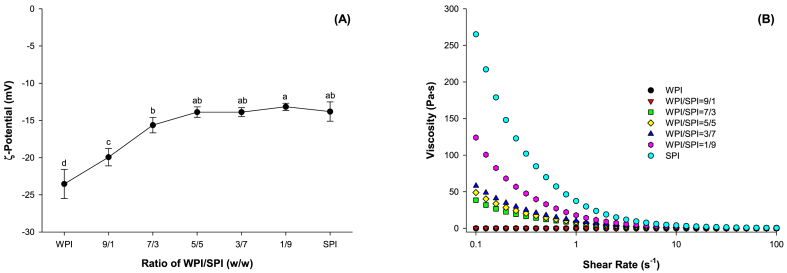
Zeta-potential (A) and viscosity (B) of emulsions prepared using mixtures of SPI and WPI at different ratios at pH 7.0 with 50 mmol/L NaCl (n = 3).

Besides the size of emulsified droplets, their *ζ*-potential affects the emulsion gels' properties (Dickinson, 2012). The *ζ*-potentials of emulsions prepared with different ratios of WPI to SPI are shown in Fig. 3A. As the SPI ratio in the protein mixtures increased from 0 to 50%, the *ζ*-potential of emulsions increased by 41% (p < 0.05). However, further increasing the proportion of SPI did not change the *ζ*-potential of the emulsions. Since the emulsions exhibited a net negative charge, the higher *ζ*-potential meant less net negative charge at the surface of emulsion droplets and excess proteins in the continuous phase. This might weaken the repulsive forces between particles and resulted in the formation of aggregates, especially for soy protein.

#### 3.1.3 Apparent viscosity

The apparent viscosity of emulsions prepared with WPI and SPI mixtures at different ratios of WPI and SPI is shown in Fig. 3B. All emulsions were a shear-thinning non-Newtonian fluid. At a low shear rate, a higher ratio of SPI to WPI in the protein mixture exhibited higher apparent viscosity. The results of CLSM (Fig. 2) confirmed that protein aggregates (green particles) increased as the proportion of SPI in the mixture increased. As little flocculation of oil droplets was observed in all emulsion samples, more protein aggregates might have led to higher apparent viscosity of emulsions. It was because higher shear was required to overcome the molecular interactions of proteins and dissociate the protein aggregates. Moreover, soy protein exhibits a higher molecular weight than whey protein. An increase in the SPI ratio could occupy more free space in the continuous phase. It might result in higher apparent viscosity.

#### 3.1.4 Protein partitioning in the emulsions

The properties of emulsion gel are not only related to the particle size and surface charge of filling droplets but also affected by the interaction between the filling droplets and the protein network formed in the matrix of gel (Dickinson, 2012). The protein distribution between the oil and aqueous phases in the emulsions prepared using mixtures of SPI and WPI at different ratios might be different. This could lead to the formation of different filling droplets and protein networks in the gel matrices.

**Fig. 4:**
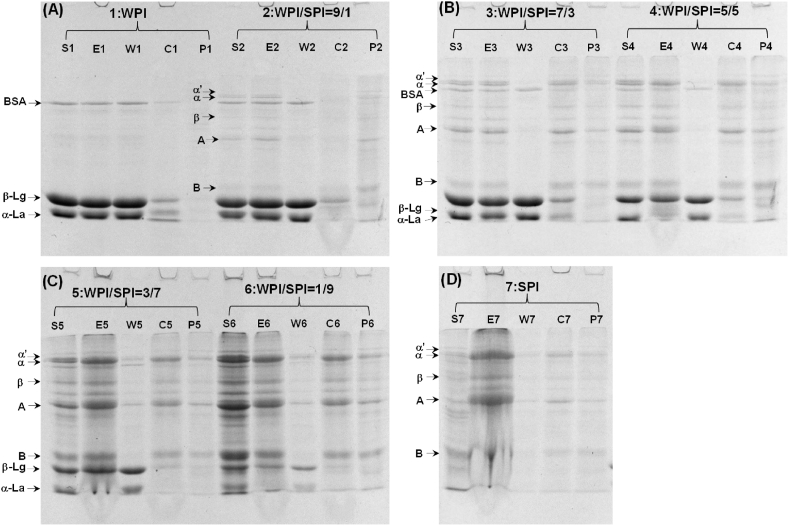
Reducing SDS-page of proteins partitioning in the emulsions prepared using a mixture of SPI and WPI at different ratios (S-protein solution, E-emulsion, C-cream, W-serum, P-precipitate).

The emulsions prepared using mixtures of SPI and WPI were separated by centrifugation and analyzed using a reduced SDS-PAGE, as shown in Fig. 4. The existence of β-conglycinin (7 S, including the subunits α′, α, β) and glycinin (11 S, including acidic and base subunits) bands from soy protein and β-lactoglobulin and α-lactoalbumin bands from whey protein were all present in the cream and sediment of the emulsions. It indicated that both whey and soy proteins were present at the interface or in the water phase of the emulsions. When the proportion of soy protein in the mixture increased, the band intensity of 7 S and 11 S of soy protein in the cream was enhanced. At the same time, the band intensity of whey proteins seemed to decrease.

The soy protein in the water phase might contain both soluble and insoluble protein complexes, as shown by the CLSM (Fig. 2). At the WPI/SPI ratios of 30:70 and 10:90, the existence of soy protein in both the serum (soluble) and sediment (insoluble) of the emulsions was identified. It is suggested that soy protein interacts with whey protein and forms soluble complexes. This is similar to the findings of Roesch and Corredig (Roesch and Corredig, 2005), who showed that soluble complexes containing whey and soy protein mixtures might form at lower concentrations of whey protein. The soy protein was mainly displayed in the sediments at the WPI/SPI ratios of 90:10, 70:30 and 50:50. This suggests that the soy protein formed the insoluble complexes in those emulsion samples.

Furthermore, a small part of whey protein was incorporated into those insoluble complexes. Similar results have been reported by McCann et al. (2018). The distribution of proteins in the water phase (serum and sediment) suggests that different ratios of WPI and SPI might form a network of the gel matrix. Those insoluble complexes might act as the filler in the final protein gels (McCann et al., 2018).

**Fig. 5:**
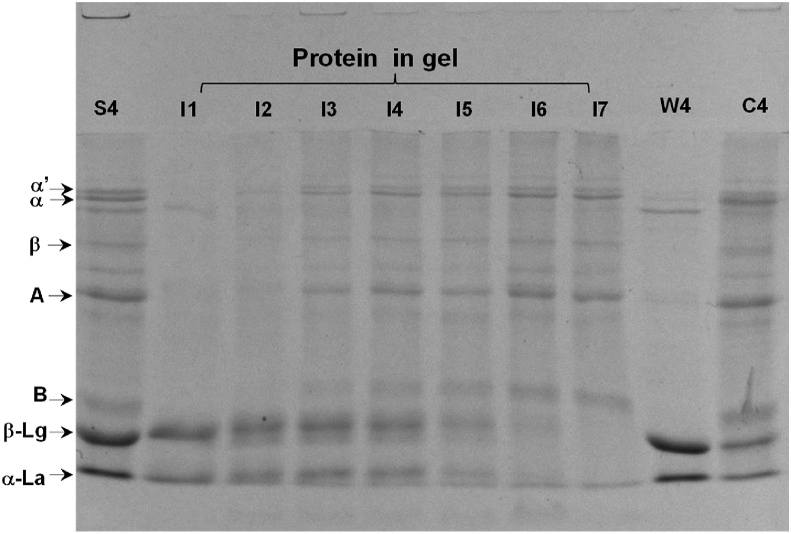
SDS-PAGE of the solution prepared by dissolving the mixing WPI and SPI emulsion gels (liquid was excluded by centrifuge) with 50 mmol β-ME and 0.5 wt% SDS (I1, I2, I3, I4, I5, I6 and I7 represented the samples prepared with WPI-SPI mixture at different ratio of 100:0, 90:10, 70:30, 50:50, 30:70, 10:90 and 0:100, respectively.).

To identify the protein incorporated into the gel matrix, protein compositions of the gel samples from which water was excluded by centrifugation were analyzed using SDS-PAGE. The co-existence of WPI and SPI in the gel matrix was displayed in Fig. 5. The subunit of α, α′, β from 7 S, A, B from 11 S, β-Lg and α-La from WPI were demonstrated to exist in the gel matrix. Increasing the proportion of SPI led to an increase in the band intensity of SPI, whereas the band intensity of WPI decreased. At high ratios of WPI to SPI, WPI seemed to be the dominant composition forming the gel network in the gel matrix according to the bands on the SDS-PAGE. This is consistent with the previous studies (Comfort and Howell, 2002; Xia et al., 2022).

Similarly, at the low ratios of WPI to SPI, SPI seemed to be the dominant composition forming the gel network in the gel matrix, which was in line with the results reported by other researchers that WPI may form the primary continuous protein network (Comfort and Howell, 2002; McCann et al., 2018). At the intermediate ratios of WPI to SPI, the co-existence of WPI and SPI was obvious, suggesting that both WPI and SPI contribute to the gel network.

### 3.2 Dynamic rheological behaviour of emulsion gels

**Fig. 6:**
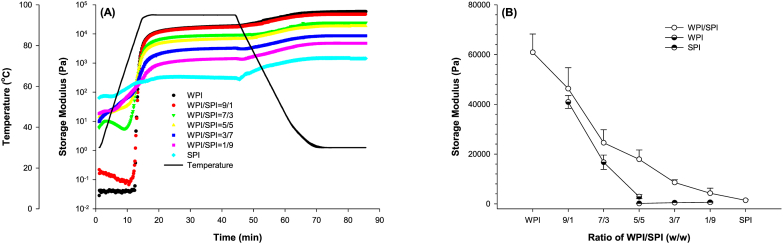
Change in storage modulus of emulsions prepared using mixed protein at different ratios of WPI and SPI during a heating and cooling cycle (A) and final storage modulus of their emulsion gels (B).

The rheological properties during gel formation are shown in Fig. 6. All samples displayed a similar trend in the change of storage modulus (G′) during the heating and cooling cycle. The G′ curve of mixed whey and soy protein emulsion gels comprised four parts. That was similar to the results of mixed whey and soy protein gels (Jose et al., 2016). The protein emulsion gel can be regarded as protein gel with oil droplets as filler and the protein network as the matrix. The changes in structure and rearrangement of protein molecules caused by heating and cooling followed the protein gel formation mechanism (Mezzenga and Fischer, 2013; Nicolai, 2019).

Since whey and soy proteins have different denaturation temperatures, mixed whey and soy proteins exhibited different rheological behaviours. As proteins are denatured at high temperatures, exposed hydrophobic amino acid residues in protein molecules would lead to intermolecular interaction between different protein molecules, forming protein aggregates during further heating and temperature-holding steps. Also, protein molecules could be cross-linked by disulphide bonds according to oxidation of the exposed free sulfhydryl group. During the following cooling step, the formation of hydrogen bonds between small aggregates leads to further protein aggregation and the formation of large protein aggregates. That resulted in rapidly increasing G′ of gels.

The ratio of WPI to SPI had a significant influence on the final G′ of mixing protein emulsion gels (Fig. 6B). The final G′ values of emulsion gels prepared with WPI were 1.47, 2.40 and 6.09 times higher than those prepared with the mixing proteins at the WPI to SPI ratio of 70:30, 50:50 and 30:70 (p < 0.05). To determine the contribution of whey or soy protein to the final G′ values of mixed protein emulsion gels, the final G′ of single protein emulsion gels prepared at whey protein or soy protein concentrations of 5.0%, 7.0% and 9.0% were analyzed (Fig. 6B). The results showed that mixing protein emulsion gels exhibited higher final G′ than single protein emulsion gels (p < 0.05). Interestingly, the final G′ of mixing protein emulsion gels at the WPI to SPI ratio of 50:50 was 2.15 times higher than that of the sum of whey and soy protein gels prepared at the protein concentration of 5.0%. It suggests that both proteins contributed to the mechanical properties of gels. Although the co-existence of WPI and SPI in the gel matrix has been evident in the SDS-PAGE (Fig. 5), it cannot be concluded that both proteins form the continuous protein gel network. Our result was consistent with acid-induced whey and soy protein composite gels with thermal pretreatment on the protein mixtures (Xia et al., 2022). At the WPI to SPI ratio of 2:2, the protein network consisted of a whey protein network and a soy protein network, according to the 3D CLSM images (Xia et al., 2022).

The thermal process leads to the formation of soy and whey protein mixture aggregates by covalent bonds (Roesch and Corredig, 2005), resulting in a composite protein gel network (McCann et al., 2018). The active filling effect of soy protein aggregates might also contribute to gel properties (McCann et al., 2018; Roesch and Corredig, 2005). Protein-emulsified oil droplets serve as fillers with a positive filling effect on the gel network (Dickinson, 2012). Furthermore, the filled oil droplets prepared with mixing protein could demonstrate a better filling effect than that with a single protein (Mao et al., 2021).

### 3.3 Hardness and water holding capacity (WHC) of emulsion gels

**Fig. 7:**
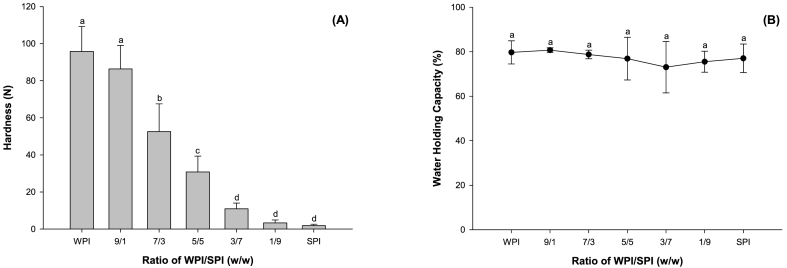
Hardness (A) and water holding capacity (B) of protein emulsion gels prepared with the mixture of WPI and SPI at different ratios.

As shown in Fig. 7A, the gel hardness displayed a similar trend as the final G'. The hardness of emulsion gels prepared with WPI was 0.73, 2.72 and 8.45 times higher than those prepared with the mixing proteins at the WPI to SPI proportion of 70:30, 50:50 and 30:70 (p < 0.05). The protein aggregates that existed before gel formation might have a negative effect on the gel texture because they occupied the space of the gel network. The gel strength of the samples exhibited a linear decrease (R^2^ = 0.99) as the WPI ratio decreased from 100% to 50%. However, the gel strength of the samples exhibited an exponential decrease as the WPI ratio decreased from 50% to 0. By contrast, replacing WPI with SPI exhibited little effect on the WHC of mixing protein emulsion gels (p > 0.05) (Fig. 7B).

### 3.4 Microstructure of emulsion gels

**Fig. 8:**
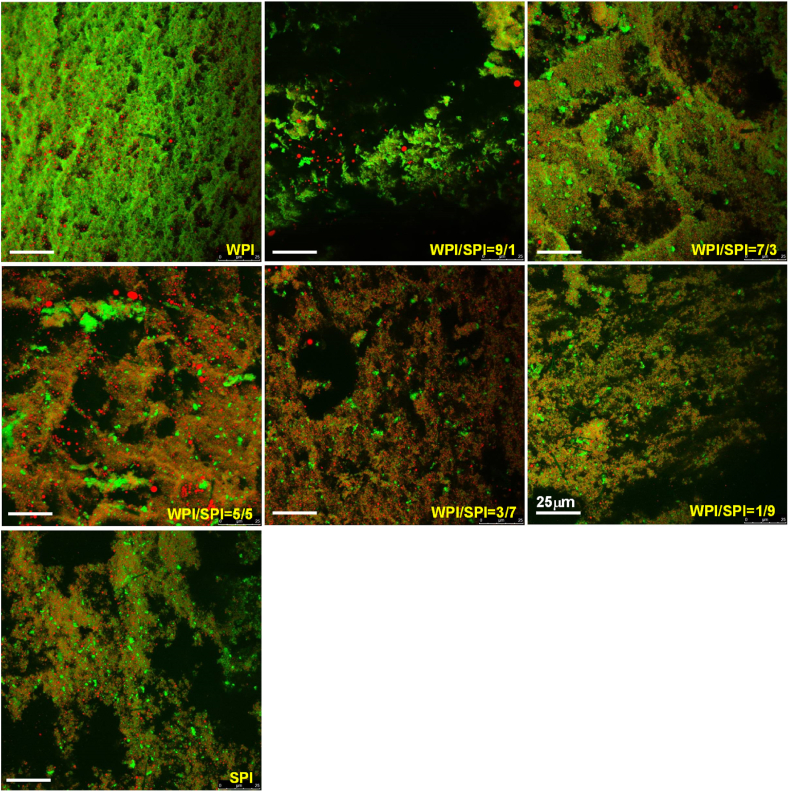
CSLM images of protein emulsion gels prepared with the mixture of WPI and SPI at different ratios (Scale bar: 25 μm).

The microstructure of mixed protein emulsion gels was analyzed using CLSM in Fig. 8. The oil droplets were red, while the proteins were green. The emulsion gels' porosity appeared to change with the WPI to SPI ratio. The size of the dark holes enlarged as the proportion of SPI in the mixture increased. It indicated that increasing the proportion of SPI in the mixture resulted in the enlargement of the pores in the gel network. The emulsion gels prepared with WPI displayed a dense protein network, exhibiting a high G′ value. The emulsion gels prepared with SPI displayed a loose protein network and a low G value. Our results were consistent with the previous work on the mixed whey and soy protein gels (Comfort and Howell, 2002; Jose et al., 2016; Roesch and Corredig, 2005).

Moreover, decreased SPI proportion raised the G′ of emulsion gels while lowering their porosity. The reason might be that the consistency of the protein network changed. The protein concentration was high in our work, so the emulsion gel matrix comprised a protein network with the embedded oil droplets.

Whey protein has a lower denaturation temperature than soy protein. Thus, it is more prone to aggregation. When the ratio of WPI to SPI was higher than 50:50, the protein network was mainly made up of whey protein because soy protein cannot form self-supported solid gels at low concentrations. Meanwhile, the existence of the soy protein in the gel matrix was displayed in SDS-PAGE in Fig. 5. It suggested that a small proportion of soy protein might work as a cross-linker of whey protein aggregates and participate in the whey protein network. It suggested that a small proportion of soy protein might work as a cross-linker of whey protein aggregates and participate in the whey protein network. Our results were consistent with previous research on whey and soy protein composite gels (Comfort and Howell, 2002; Roesch and Corredig, 2005). That might lead to the increase in the G′ of emulsion gels prepared with the mixture of WPI and SPI at the ratio of 70:30 when compared with that prepared with a single protein of WPI at the same whey protein concentration of 7.0%.

On the other hand, the CLSM images of emulsion gels with SPI showed the existence of some protein particles, which might be the soy protein aggregates (Comfort and Howell, 2002; Roesch and Corredig, 2005). These soy protein aggregates might act as the active filler in the emulsion gels and improve the G′ of emulsion gels. With the increase in the concentration of SPI, a part of soy protein might occupy the space between the small aggregates of whey protein and hinder them from forming the network of whey protein.

At the WPI/SPI ratio of 50:50, the protein network in the gel matrix could be made up of both WPI and SPI. The CLSM displayed both oil droplets and soy protein aggregates that could act as the active filler, resulting in a higher G′ of the emulsion gel formed with the mixture than that of the gel formed by a single protein (Fig. 6B). When the ratio of WPI to SPI was lower than 50:50, the protein network was mainly made up of large soy protein aggregates. as shown in CLSM. The existence of WPI, which mainly existed in the water phase of emulsions, seemed to cross-link those aggregates together and reduce the porosity of the protein network.

### 3.5 Gastric digestion of mixed whey and soybean protein emulsion gels

**Fig. 9:**
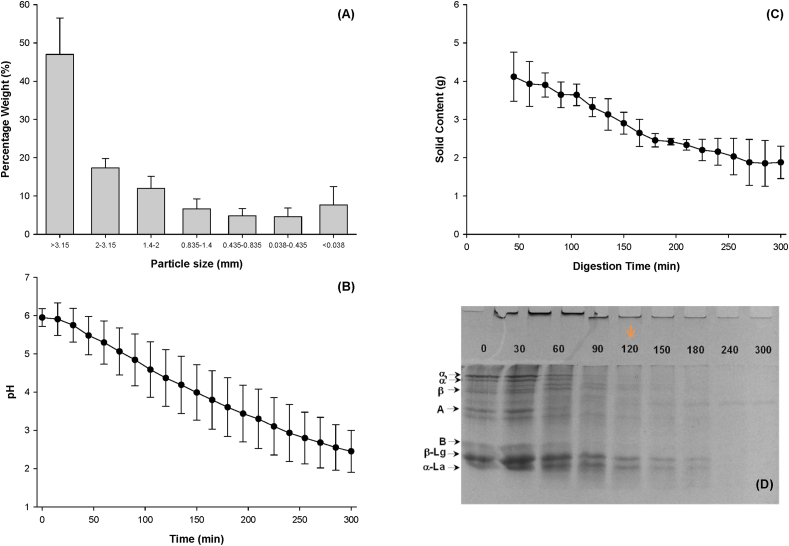
Particle size distribution of bolus of mixed protein emulsion gels after simulated oral digestion; pH change (B), solid content (C) and SDS-Page (D) of emptied digesta of mixed protein emulsion gels (WPI:SPI = 50:50) during gastric digestion.

Mixed whey and soybean protein emulsion gels with a protein ratio 50:50 were selected for digestion. The protein emulsion gels were broken down using a simulated oral process. The particle size distribution of the gel boluses was characterized by sieving, and particle size distribution in weight is shown in Fig. 9. Sixty-four per cent of the gel bolus has remained above the sieve with a size of 2 mm. It was consistent with the research results (Luo et al., 2020) that soft gels contained more large bolus particles during the oral process.

After the oral process, the gel bolus was loaded to the HGS to mimic gastric digestion. The pH of the gastric digesta at the beginning of gastric digestion rose to 5.95 when gel bolus was mixed with the SGF under fasting stomach conditions. Then, the continuous addition of SGF decreased the pH of the gastric digesta. The pH of the gastric digesta dropped to 2.45 at the end of the digestion.

The degradation of proteins was indicated using the SDS-PAGE, as shown in Fig. 9D. During the digestion time of 120 min, the band intensity of both soy protein and whey protein reduced with the increased digestion time. After 120 min, almost all the bands of soy protein disappeared in the SDS-PAGE while a portion of whey protein still existed. This suggested that pepsin hydrolyzed soy protein faster than WPI under these conditions. For soy protein, the 11 S (glycinin) was more prone to be hydrolyzed than the 7 S (β-conglycinin). Little bands of subunits A and B were seen on the SDS page after 90 min of digestion, whereas the bands of subunits α, α′ and β disappeared after 120 min of digestion. This result agreed with the previous study in which glycinin released more peptides than β-conglycinin during gastric digestion (Wang, et al., 2023). β-Conglycinin was shown to survive to same content during gastric digestion (Amigo-Benavent, et al., 2011). Regarding the results of protein not in the gels (data was not shown), the amount of subunits A and B that did not take apart in the gel matrix was more than that of subunit α α′ and β. That might lead to the faster digestion of 11 S.

The bands of β-Lg and α-La disappeared after 240 min of digestion. After 300 min of digestion, most large aggregates were digested, as indicated by the loss of the protein bands. The whey protein network in the gel matrix might be surrounded by soy protein (Comfort and Howell, 2002; Xia et al., 2022) in certain positions because soy protein was prone to form protein aggregates. The CLSM image showed that protein aggregates were incorporated into the protein network. The whey protein in the gel matrix might have been protected from the action of pepsin before the soy protein was exhausted. Moreover, soy protein partitioning at the surface of oil droplets might also lead to faster digestion of SPI.

## 4 Conclusions

The mixed protein emulsion gels were prepared at different WPI to SPI ratios. The results showed that the ratios of WPI to SPI significantly affected the mechanical and rheological properties and microstructure of mixed protein emulsion gels. Partial replacement of WPI with SPI incorporated soy protein into the gel network, leading to an inconsistent gel network and high gel porosity. The hardness and final storage modulus decreased with the increasing ratio of SPI. Both β-lactoglobulin and α-lactalbumin from WPI and 7 S and 11 S from SPI contributed to the matrix of the composite protein emulsion gels. Soy protein was more sensitive to pepsin than whey protein during gastric digestion. Mixed soy and whey protein emulsion gels could be used to develop food products with different characteristics.

## CRediT authorship contribution statement

**Yu Cheng:** Investigation, Data curation, Writing – original draft. **Aiqian Ye:** Conceptualization, Supervision, Writing – review & editing, Funding acquisition. **Harjinder Singh:** Supervision, Writing – review & editing, Funding acquisition.

## Declaration of competing interest

The authors declare that they have no known competing financial interests or personal relationships that could have appeared to influence the work reported in this paper.

## Data Availability

Data will be made available on request.
